# Anti-Inflammatory Effects of Algae-Derived Biomolecules in Gut Health: A Review

**DOI:** 10.3390/ijms26030885

**Published:** 2025-01-21

**Authors:** Alessia Brizzi, Rosaria Margherita Rispoli, Giuseppina Autore, Stefania Marzocco

**Affiliations:** 1 Department of Pharmacy, University of Salerno, Via Giovanni Paolo II 132, 84084 Fisciano, Salerno, Italy; abrizzi@unisa.it (A.B.); r.rispoli20@studenti.unisa.it (R.M.R.); autore@unisa.it (G.A.); 2 Ph.D. Program in Drug Discovery and Development, University of Salerno, Via Giovanni Paolo II 132, 84084 Fisciano, Salerno, Italy

**Keywords:** algae, biomolecules, gut inflammation, IBD

## Abstract

Under physiological conditions, the inflammatory response acts as a biological defense against tissue damage or infection, and is rapidly resolved once the infection is cleared. However, chronic inflammatory diseases, including inflammatory bowel disease (IBD), have become increasingly widespread in the last decades, placing a burden on the quality of life of affected people and on healthcare systems worldwide. Available drug therapies are often ineffective due to the chronic nature of these diseases, and prolonged administration of drugs can result in severe side effects for the patient or a lack of efficacy. In addition, there is the growing problem of bacterial resistance to synthetic antibiotics. Together, these factors have led to a strong research focus on the discovery of natural products capable of treating IBD. Recently, there has been a growing interest in compounds derived from marine sources, mainly algae, due to their bioactive secondary metabolites with anti-inflammatory properties well known in the literature. Based on this evidence, this review aimed to evaluate the anti-inflammatory potential of algae-derived biomolecules in IBD. In particular, interesting species from green algae (e.g., *Chlorella vulgaris* and *Ulva pertusa*), brown algae (e.g., *Macrocystis pyrifera* and *Ecklonia cava*) and red algae (e.g., *Porphyra tenera* and *Grateloupia turuturu*) are included in this review due to their proven anti-inflammatory properties. For this purpose, an extensive literature search was conducted using several databases. The results suggest that both macroalgae and microalgae have remarkable potential for IBD therapy due to the anti-inflammatory and antioxidant activities of their bioactive compounds. However, while the preclinical evidence is encouraging, further and long-term clinical studies are needed to better understand their mechanisms of action in order to determine the true efficacy of marine algae in the treatment of IBD.

## 1. Introduction

Inflammatory bowel disease (IBD) is an immune-mediated disease characterized by chronic inflammation of the gastrointestinal tract resulting in progressive damage to the affected intestinal tissue [1]. IBD can be clinically classified as Crohn’s disease (CD) or ulcerative colitis (UC) based on factors such as symptoms, specific site of the damage, and histopathologic features. Symptoms of UC include rectal bleeding, urgency, diarrhea, and abdominal cramping, along with fatigue and fever [2]. While UC can lead to chronic inflammation and superficial ulcers in the colon, CD is characterized by non-continuous inflammation that can affect any part of the gastrointestinal tract and that is frequently associated with granuloma formation [3]. Patients with CD may present with symptoms such as abdominal pain, fever, diarrhea with blood or mucus, and indicative signs of intestinal obstruction [4]. It is a chronic inflammatory condition often associated with metabolic syndrome, cardiovascular disease, atherosclerosis, chronic obstructive pulmonary disease, Parkinson’s disease, psoriatic arthritis, osteoporosis, and psychological and psychiatric disorders [5]. Given its chronic nature and potentially debilitating effects on patients’ quality of life, IBD has received increasing attention over the years, also considering that UC and CD affect millions of people worldwide, with a disease prevalence in the United States that is expected to increase by 229% by 2030, based on the number of diagnoses in 2010 [6].

The etiology of IBD is not fully understood, but it is currently hypothesized that the abnormal immune response may be related to the dysregulation of both the innate and adaptive immune systems. Persistent inflammation, along with oxidative stress, contributes to inappropriate immune responses and to an imbalance in the gut microbiota. These factors lead to altered levels of pro- and anti-inflammatory signals, exacerbating inflammation and disrupting both host mechanisms and factors in the intestinal lumen [7,8]. Consequently, extensive research over the years has been focused on the identification of different inflammatory pathways, which has allowed the development of the currently most used therapeutic strategies aimed at the management of inflammation. One of the most studied pathways involves the tumor necrosis factor alpha (TNF-α), a pro-inflammatory cytokine. Numerous anti-TNF-α drugs have been developed in order to attenuate the inflammatory response, although many patients either do not respond initially or may lose their response over time [9]. In addition, studies in both humans and animal models have demonstrated a dysregulation of macrophage-mediated immune responses to the gut microbiota, which disrupts homeostasis and results in reduced immune tolerance and elevated pro-inflammatory cytokines. As a result, the treatment of moderate and severe cases of IBD focuses on targeting cytokines, among which IL-12 and IL-23 stand out [7].

On the other hand, severe dysbiosis of gut microbiota is observed in IBD patients [10]. This imbalance is often characterized by a decrease in bacterial biodiversity, as well as a reduction in the population of bacteria with anti-inflammatory properties and an increase in the population of bacteria capable of stimulating pro-inflammatory activity [11]. These observations have led to the hypothesis that treating dysbiosis with drugs enriched in those bacteria that are less abundant in the microbiota of IBD patients and that may produce anti-inflammatory substances may reduce intestinal inflammation [12].

In addition to intestinal inflammation, oxidative stress plays an interesting role in IBD, as the inflamed intestinal mucosa is associated with signs of hypoxia and increased oxidative stress [13]. Inflammation consumes a large amount of oxygen and produces harmful free radicals. Studies have shown that IBD patients have elevated levels of reactive oxygen species (ROS), while they may have reduced levels of antioxidant molecules in their intestinal mucosa, conditions that contribute to disrupting homeostasis and exacerbating tissue damage [14].

In fact, the first-line treatment for mild to moderate cases of CD and UC consists of the continuous administration of corticosteroids, aminosalicylates, and antibiotics [15]. Recently, however, algae-derived metabolites have become increasingly important in the nutraceutical field as they show promising results for various pharmacological applications due to their antioxidant, anti-inflammatory, anti-hypertensive, anti-diabetic and cardioprotective effects [16].

Algae are aquatic photosynthetic organisms known for their great potential as a source of affordable bioactive compounds. They are particularly suitable for various applications in the pharmaceutical, food, and cosmetic fields because algae are capable of synthesizing biomolecules in a short growth period and through a simple and environmentally friendly cultivation method [17]. Algae can be classified into multicellular (seaweeds or macroalgae), unicellular, or colonial forms (including microalgae and cyanobacteria). Marine algae are further divided into major phyla, based on their pigments: Chlorophyta (green algae), Rhodophyta (red algae), Phaeophyta (brown algae) and Cyanophyta (blue-green prokaryotic algae) [18].

The selection of algae as a target species for the study of anti-inflammatory effects has received considerable attention in scientific research due to the wealth of compounds they produce, which have potential therapeutic properties, as demonstrated by studies on algae such as *Chlorella vulgaris* and *Ulva pertusa* [19,20,21]. In fact, the bioactive molecules produced by algae have shown promising effects in modulating immune responses and reducing inflammation. Some of these bioactive substances are carbohydrates, proteins, fatty acids (FAs), polyunsaturated fatty acids (PUFAs), minerals, amines, amides, antioxidants, and pigments. A notable example of algae’s beneficial properties is given by fucoidans, polysaccharides typically extracted from brown algae, which exhibit antioxidant, antiproliferative, antitumor, and anti-inflammatory properties, leading to promising results for IBD treatment [22]. Effects of fucoidans on colitis have been tested in several studies after extracting fucoidans from algae such as *Undaria pinnatifida* and *Macrocystis pyrifera*, which showed anti-inflammatory and antioxidant activities [23,24]. Oligo- and polysaccharides isolated from algae of the genus *Laminaria* have also been shown to reduce the intestinal inflammatory response [25]. Another interesting compound is caulerpin, produced by algae of the genus *Caulerpa*, which can modulate the release of pro- and anti-inflammatory cytokines [26]. Tannins produced by algae can also modulate the inflammation, as shown by studies of DSS-induced colitis models treated with eckol and dieckol isolated from the alga *Ecklonia cava* [27,28]. In addition, brown algae such as *Sargassum macrocarpum*, *Dictyopteris undulata*, and *Turbinaria ornata* can ameliorate inflammation-induced oxidative stress and the balance of pro- and anti-inflammatory cytokines [29,30,31]. The role of polyphenols produced by algae (e.g.: *Porphyra tenera*) is also of great interest, considering their anti-inflammatory effect on a DSS-induced colitis model, as well as PUFAs extracted from red algae, such as *Grateloupia turuturu*, which can reduce oxidative stress [32,33].

In this context, the main objective of this review was to explore and report on the therapeutic potential of various marine algae species, with special focus on their antioxidant and anti-inflammatory activities, which could be useful in the pharmacological context for the treatment of IBD.

## 2. Materials and Methods

To identify relevant studies, a comprehensive literature search was conducted using the PubMed (MEDLINE), Google Scholar and Scopus databases, which were queried using keywords such as “algae”, “seaweed”, “inflammatory bowel disease”, “IBD”, “intestinal inflammation”, and “gut inflammation”. Data extracted from relevant studies have been selected based on publication relevance, research methodology and results, statistical significance, and publication date, up to 10 October 2024. In addition, a manual search of the reference lists of key studies was conducted to identify additional relevant data. A total of 100 publications was selected, including systematic reviews, research articles, and meta-analyses.

## 3. IBD Pathophysiology

### 3.1. Characterization of IBD Patients’ Intestinal Mucosa

**Figure 1:**
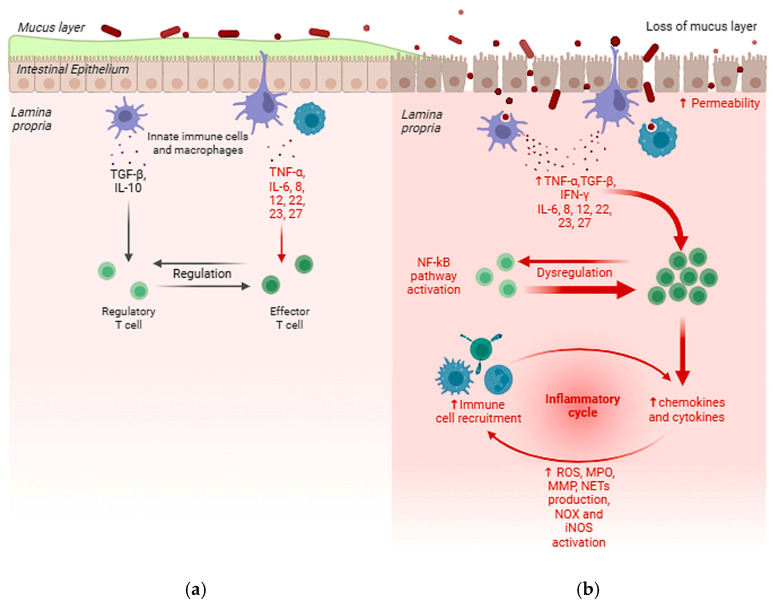
A healthy intestinal epithelium is characterized by a normal mucus layer that prevents excessive bacterial infiltration and its immune regulation functions properly (**a**). On the other side, the typical gut affected by IBD produces less mucus, and it is characterized by breaches in the epithelium, leading to increased permeability and dysregulation of immune cell recruitment due to increased levels of chemokines and cytokines, which cause a chronic inflammatory response, enhanced by the increased production of ROS, MPO, MMP and NETs, as well as the increased NOX and iNOS activation (**b**). ↑ = increased.

IBD is typically characterized by disruption of the intestinal epithelial barrier (Figure 1) along with a chronic inflammatory response due to persistent mucosal damage from infectious agents, chemical substances, or metabolic dysbiosis [34,35]. The intestine is organized into a series of projections called “villi” and invaginations known as “crypts of Lieberkühn”. The intestinal epithelium is composed of a monolayer of epithelial cells tightly connected by junctions, along with interspersed immune cells. The two primary functions of the intestinal epithelium are: (a) the absorption of nutrients and (b) forming a physical barrier around the intestinal lumen. Both the epithelium and other non-immune components of the intestine play a significant role in the pathophysiology of IBD [36]. The intestinal mucosa contains goblet and Paneth cells that produce mucus and antimicrobial peptides, respectively, within the lumen. A significant decrease in goblet cell numbers has been associated with a reduction in mucus thickness in CD, while abnormal mucus composition has been observed in UC [37]. Beneath the lamina propria, the epithelium consists of stromal cells such as fibroblasts, myofibroblasts, and perivascular pericytes, which may contribute to the exacerbation of UC by producing chemokines [38] and are involved in the formation of fibrosis [37].

### 3.2. Innate Immune System Activation

When the intestinal barrier is damaged, a number of chemotactic substances are released, including cytokines (such as IL-1β, IL-6, and TNF-α), chemokines, and growth factors. These substances attract innate immune cells (IIC) such as neutrophils, monocytes, macrophages, and dendritic cells, from the bloodstream to the site of inflammation, where they can identify pathogen-associated molecular patterns (PAMPs) and damage-associated molecular patterns (DAMPs) released by damaged or dying cells through their interactions with pattern recognition receptors (PRRs) such as toll-like receptors (TLRs) and NOD-like receptors (NLR). In addition, these patterns can be recognized by non-immune cells, such as intestinal epithelial cells (IECs) and myofibroblasts. This pathway activates the innate immune system, leading to non-specific responses against microorganisms by producing cytokines and chemokines, triggering the complement cascade and phagocytosis, or by stimulating adaptive immunity through antigen presentation [39]. In addition, nuclear factor-κB (NF-κB) can be activated in response to various stimuli. NF-κB is a group of transcription factors that plays a critical role in promoting the expression of several pro-inflammatory genes and also regulates the activation and differentiation of innate immune cells and inflammatory T cells. Indeed, the development of a number of inflammatory diseases can be correlated with abnormal activation of the NF-κB pathway [40].

### 3.3. Neutrophils in IBD Pathogenesis

Neutrophils play a critical role in eliminating microorganisms through processes such as phagocytosis, ROS production, and the release of neutrophil extracellular traps (NETs). When the intestinal barrier is compromised, as it is in IBD patients, NETs can prevent the proliferation of microorganisms by trapping them in an antimicrobial environment. In addition, neutrophils also contribute to the healing of the damaged intestinal mucosa [41].

An essential step in NET production is histone citrullination, a process facilitated by increased levels of protein arginine deiminase 4 (PAD4) [42]. Neutrophils in colon samples from UC patients produce more NETs in response to TNF-α treatment, accompanied by a significant abundance of PAD4 and citrullinated histone H3 (CitH3) in UC colon tissue [43]. In a healthy gut, neutrophils undergo apoptosis after completing their functions, allowing the resolution of the inflammation and the restoration of tissue homeostasis. However, compared to healthy individuals, IBD patients show increased neutrophil activity, which can be found by analyzing the levels of myeloperoxidase (MPO), a lysosomal protein of neutrophils, which is considered an interesting fecal biomarker in IBD diagnosis [44]. This high neutrophil activity compromises the intestinal mucosal barrier and may worsen the symptoms of IBD [41]. Indeed, studies in mice with dextran sodium sulfate (DSS)-induced colitis have shown an accumulation of NETs in colonic samples, associated with the disruption of tight junctions, epithelial cell apoptosis, and increased bacterial translocation. In addition, a reduction in NET formation may have beneficial effects against colitis and may inhibit the increased production of pro-inflammatory factors associated with IBD, while the accumulation of NETs enhances plasmatic production of TNF-α and IL-1β in plasma by signaling through the ERK1/2 pathway [45]. The increased release of pro-inflammatory cytokines produced by endothelial cells further compromises the epithelial barrier by recruiting monocytes and additional neutrophils into the inflamed tissue [46]. In addition, neutrophils directly cause intestinal tissue damage by releasing proteases, such as matrix metalloproteinases (MMPs) and neutrophil elastase, and by altering membrane properties through the release of ROS [42].

### 3.4. Oxidative Stress in IBD Pathogenesis

The inflammatory process is associated with increased levels of ROS produced by immune cells located in the damaged area. Major ROS metabolites produced include superoxide anion (O_2_^−^), hydroxyl radical (**·**OH), hydroperoxyl radical (HO_2_^•^), nitric oxide (NO), and singlet oxygen (^1^O_2_). ROS upregulate genes involved in innate and adaptive immune responses in order to amplify mucosal inflammation [47]. Indeed, excessive levels of ROS can impair the functionality of cytoskeletal proteins, alter tight junctions, and lead to increased intestinal permeability. This disruption of the intestinal epithelial barrier exacerbates mucosal inflammation [14]. In this context, inflammatory cytokines induce IECs, neutrophils, and macrophages to produce O_2_^−^ and NO by activating NADPH oxidase (NOX) and inducible nitric oxide synthase (iNOS) [47].

In addition, during the active phase of IBD, there is a metabolic transition toward hypoxia associated with mucosal inflammation. This shift activates the transcription factor hypoxia-inducible factor (HIF)-1, which is increasingly stabilized in the inflamed areas. In this context, HIF-1 provides a protective role against inflammation and IBD by promoting the expression of several genes, capable of enabling intestinal epithelial cells to function as an effective barrier [48]. Nevertheless, oxidative damage has been observed in the intestinal tissue of individuals with CD [49]. Indeed, ROS reduce the accumulation of antioxidant defenses within host cells, leading to cellular and intestinal tissue damage. Furthermore, in inflammatory situations in CD patients, low concentrations of antioxidant vitamins A, C, E, and beta-carotene further contribute to the oxidative stress condition [50].

### 3.5. Macrophages in IBD Pathogenesis

IBD patients have a high number of pro-inflammatory macrophages, and increased expression of pro-inflammatory molecules such as TNF-α, IL-1β, IL-6, and iNOS [51]. Under healthy conditions, macrophages exert microbicidal activity in host defense by discriminating between harmful and commensal bacteria through PRRs. This recognition allows them to produce cytokines that are typically secreted by T cells, natural killer cells, or antigen-presenting cells (APCs) [52]. In the context of IBD, mucosal epithelial cells produce IL-8 and transforming growth factor (TGF)-β, which are involved in regulating the recruitment of Ly6Chigh inflammatory monocytes through a C-C motif chemokine ligand 2 (CCR2)-dependent mechanism, as a result of the local release of PAMPs and DAMPs in the damaged tissue [53]. Consequently, the recruitment of Ly6Chigh monocytes enhances the expression of TLR2 and NOD2, which promotes their differentiation into pro-inflammatory effector cells [54] and allows them to produce pro-inflammatory cytokines [52].

In addition, IL-34 is overproduced in IBD patients. This is interesting because IL-34 induces macrophage maturation [55] and collagen synthesis by intestinal fibroblasts [56]. Macrophages may play a role in gut fibrosis in IBD patients by enhancing myofibroblast-mediated fibrosis through the secretion of TGF-β1, connective tissue growth factor (CTGF), and fibroblast activation protein (FAP) [57]. However, the production of IL-4 by T cells or granulocytes can limit intestinal fibrosis by stimulating macrophages to promote gut tissue healing and myofibroblast senescence by degrading the extracellular matrix (ECM) [58].

In addition, regulatory macrophages, known for their anti-inflammatory properties, are produced in response to various stimuli, including the release of IL-10, glucocorticoids, and apoptotic cells [52]. It has been observed that IL-10 can alleviate symptoms of colitis when the gut microbiota is stimulated to increase the number of macrophages capable of producing IL-10 [59].

### 3.6. Current Treatment

Traditional therapeutic approaches focus on managing inflammation, and they remain the first-line treatment for achieving clinical remission in IBD. Despite the introduction of new biological agents and/or small molecules for the treatment of IBD, conventional drugs are still widely used due to their efficacy, reliability, and moderate cost for the treatment of IBD. Among them, the most commonly adopted therapies are based on 5-aminosalicylates (5-ASA), corticosteroids, immunomodulators, and anti-TNF-α agents [60]. For example, therapies based on 5-ASA administration are commonly used for UC treatment, but their side effects include fever, vomiting, paradoxical diarrhea, muscle pain, and abdominal pain. In extreme cases, 5-ASA-based medications may cause serious side effects, such as hepatotoxicity, pancreatitis, interstitial nephritis, pleuritis, or pericarditis [61].

Similarly, corticosteroids are potent drugs that act quickly to induce remission in cases of moderate to severe IBD. They rapidly reduce inflammation by decreasing intestinal permeability and decreasing the production of TNF-α [62]. Nevertheless, prolonged use of steroids for more than three months is not recommended, as it has not been shown to effectively prevent exacerbations [63] and is associated with numerous short- and long-term side effects, including hyperglycemia, hypertension, electrolyte imbalance, and increased risk of infections, osteoporosis, avascular necrosis, and adrenal insufficiency [64].

Patients who are dependent on corticosteroids respond well initially, but they often experience a relapse quickly. Similarly, patients who do not respond to corticosteroids at all should consider minimizing prolonged unnecessary corticosteroid exposure by using immunomodulators [65]. Traditional immunomodulators include azathioprine (AZT), 6-mercaptopurine (6-MP) and methotrexate (MTX). Their effectiveness is often limited when used at lower doses. This is particularly true for patients who cannot tolerate AZT due to dose-related side effects such as nausea, pancreatitis, hepatotoxicity, and leukopenia. These patients may benefit from switching to 6-MP therapy, which can provide similar efficacy and tolerability at half the dose of AZT [66], while MTX has been shown to be ineffective for long-term maintenance in UC [67]. In addition, patients treated with immunomodulators should regularly be screened for latent tuberculosis (LTB), hepatitis B virus (HBV), hepatitis C virus (HCV), and human immunodeficiency virus (HIV), as immunomodulator treatment is associated with an increased risk of infection [68].

In addition, the use of a combination of an immunomodulator and an anti-TNF-α agent reduces the immunogenicity and efficacy of anti-TNF-α therapies in IBD patients [69]. Anti-TNF-α agents are also used in patients with moderate to severe IBD who do not respond to corticosteroids or immunomodulators [70]. However, the use of anti-TNF-α agents is also associated with several serious side effects. For example, they are associated with an increased risk of melanoma and hepato-splenic T cell lymphoma, as well as an increase in infectious events in IBD patients [71]. Therefore, routine screening for LTB, HBV, HCV, and HIV is important in patients treated with an anti-TNF-α agent prior to treatment initiation [72].

## 4. Potential of Algae-Derived Biomolecules in IBD Treatment

### 4.1. Natural Compounds in IBD Studies

The low remission rates and significant side effects associated with traditional IBD drugs have led to a growing interest in exploring new therapeutic approaches. Plant matrices are important sources of various types of bioactive compounds with antioxidant properties. These substances are widely used as ingredients in functional foods and nutraceutical products [73]. Plant-derived compounds have also attracted attention in the treatment of IBD because of their diverse biological effects, as evidenced by their widespread use in traditional medicine, which has historically played a crucial role in drug discovery [74]. An example is given by *Astragalus membranaceus* dried root extract, also known as *Astragali radix*, which is widely used in traditional Chinese medicine for its biological effects [75]. It has been reported that *Astragali radix* significantly attenuates the inflammatory response in an intestinal epithelial cell line (IEC-6), reducing the amount and release of TNF-α, iNOS and ROS, while it may be able to increase the expression of cytoprotective antioxidant molecules [76]. The anti-inflammatory and antioxidant properties of these natural compounds were confirmed by Márquez-Flores et al. (2016) in their study on apigenin, which showed beneficial effects on DSS-induced colitis in mice [77]. Another example is the stem, bark and latex of *Himatanthus sucuuba* (Woodson), which have long been used by Amazonian communities for their anti-inflammatory and anti-ulcer properties [78]. The beneficial biological effects of *Himatanthus sucuuba* are attributed to its major bioactive component, plumericin, which can enhance adhesion molecule expression and actin cytoskeleton rearrangement in IEC-6 cells [79]. Furthermore, both in vitro and in vivo studies have demonstrated that baicalein, a bioactive flavone extracted from the root of *Scutellaria baicalensis* Georgi, is able to alleviate the effects of IBD [80]. Also *Punica granatum* L. juice extract has been proposed as an adjuvant in the treatment of intestinal inflammatory states and oxidative stress due to its inhibitory effect on pro-inflammatory factors [81].

In this context, the use of algae for therapeutic purposes has aroused great interest, leading to several investigations in the last 10 years (Table 1). Indeed, they are an important and renewable source of bioactive compounds [82], which have many beneficial effects on human health, such as improving the gut microbiota, reducing inflammation, and alleviating pain [83]. Algae have shown promising potential in promoting gut health due to their rich nutritional profile and bioactive compounds. Some of these bioactive compounds include polysaccharides, proteins, sterols, terpenoids, polyphenols, cyclic polysulfide compounds, and more [84].Algal-derived compounds could be integrated into biofunctional foods or dietary supplements to ameliorate the inflammatory state; in fact, they could be exploited to modulate the gut microbiota as a source of prebiotics and to improve the gut barrier function, and finally, to balance the immune response.

### 4.2. Anti-Inflammatory Activity of Chlorophyta (Green Algae) Extracts

The anti-inflammatory properties of green algae have been investigated in several studies. One of the most recent of them has demonstrated the protective effects of dried and powdered *Chlorella vulgaris* hot water extract in DSS-induced colitis in mice, orally administered in a dose equivalent to 2 g/kg of the body weight, which increased the levels of regulatory T cells [19].

**Figure 2:**
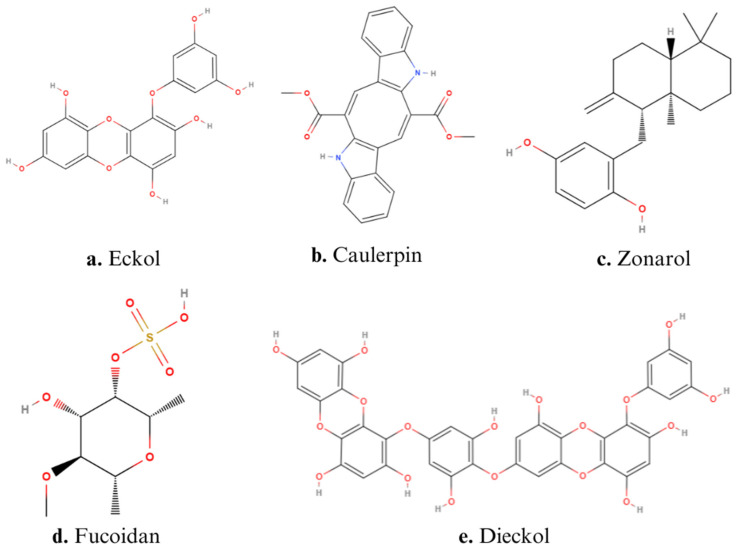
Structures of main algae-derived bioactive compounds: (**a**) Eckol, (**b**) Caulerpin, (**c**) Zonarol, (**d**) Fucoidan, (**e**) Dieckol.

Lucena et al. demonstrated the anti-inflammatory effect of caulerpin (Figure 2b), a ligand bis-indole alkaloid extracted from green algae belonging to the genus *Caulerpa*. Caulerpin can exert several effects in the organism, such as antitumor, anticancer activity, antimicrobial, cytotoxic, antidiabetic, and antiviral activity. Moreover, studies confirmed the anti-inflammatory properties of caulerpin [85]. Caulerpin inhibited pro-inflammatory responses in DSS-induced colitis mice (orally administered in a dose equivalent to 40 and 4 mg/kg of the body weight) by reducing the levels of TNF-α, IFN-γ, IL-6, IL-17, and NFκB p65, while increasing the levels of IL-10. In addition, caulerpin alleviated the colon shortening and damage [26].

The green seaweed *Ulva pertusa* has attracted great attention due to its anti-inflammatory effects. A study on its extract as a treatment for a DNBS-induced colitis mouse model showed positive results on colonic immune cell infiltration, highlighting that *Ulva pertusa* treatment can have beneficial effects by decreasing pro-inflammatory mediators such as cyclooxygenase (COX-2) and iNOS, while modulating the NF-κB pathway in mice with colitis. Indeed, the administration of *Ulva pertusa* extract facilitated the interaction between mast cells and NF-κB signaling, leading to a decrease in the release of pro-inflammatory factors while enhancing the anti-inflammatory effects of IL-4 by increasing its levels. These positive results are correlated with the activity of *Ulva pertusa* in oxidative stress. In fact, treatment with this green alga showed significant efficacy in reducing NO production and, consequently, in attenuating the activation of nitrosative stress in DNBS-induced colitis. Moreover, *Ulva pertusa* showed a significant improvement among oxidative stress indicators, such as superoxide dismutase (SOD), catalase (CAT), glutathione (GSH) and malondialdehyde (MDA) levels. Furthermore, *Ulva* extract water-soluble compound therapy also showed an anti-apoptotic effect on UC by inhibiting the apoptosis pathway, reversing the cell death process and promoting the maintenance of homeostasis in intestinal epithelial cells when orally administered in a dose equivalent to 10–50–100 mg/kg [20]. Another recent study confirmed the anti-inflammatory effect of *Ulva pertusa* by analyzing serum from a DNBS-induced colitis model (oral administration of a dose equivalent to 50–100 mg/kg of the body weight), which showed a significant decrease in pro-inflammatory biomarkers, including IL-6, IL-17, and IL-23, and a rebalancing of the anti-inflammatory IL-10. By preventing prolonged immuno-inflammatory responses, the green alga extract also showed an important analgesic effect on the abdominal pain induced by inflammatory processes of the colon [21].

There are other green algae involved in IBD studies, which are not directly related to the anti-inflammatory aspect. One example is given by *Enteromorpha clathrata*, an edible seaweed, whose beneficial effect on UC was first demonstrated by Ma et al. (2022). In their study, *Enteromorpha clathrata* polysaccharide (ECP) improved the UC condition in DSS-fed mice in several aspects: it reduced the incidence of intestinal bleeding, ameliorated mucosal damage, and improved the gut microbiota composition by increasing the abundance of probiotic bacteria *Parabacteroides* spp., which promoted increased production of SCFAs [86].

### 4.3. Anti-Inflammatory Activity of Phaeophyta (Brown Algae) Extracts

Many studies have been conducted on anti-inflammatory compounds of brown algae. An example is given by the alginate oligosaccharides (AOS), which are bioactive compounds with anti-inflammatory and antioxidant properties [87]. A study on the plasma metabolomic profile of a model of soybean-induced intestinal inflammation in zebrafish (*Danio rerio*) showed that a diet composed of soybean meal enriched with algal β-(1, 3)-glucan or a low molecular weight fraction of AOS extracted from species of the genus *Laminaria* (administered in a dose equivalent to 5% of the body weight) was capable of reducing the gut inflammation, while an intestinal transcriptomic analysis demonstrated that this type of diet can reduce the expression of genes related to the inflammatory response [25].

Several studies have investigated the effects of polysaccharides extracted from brown algae, particularly the anti-inflammatory effects of fucoidan, a sulfated polysaccharide extracted from brown seaweed (Figure 2d). The structure of fucoidan contains a backbone of sulfated fucans, which in some species shows branching of sugars, fucose, or uronic acid. The branching and the heterogeneous biochemical properties of the compound make it difficult to study the molecule as a whole [88]. An example of fucoidan activity is given by the aqueous extract solutions (AESs) isolated from *Undaria pinnatifida*, whose composition is rich in polysaccharides, which showed interesting beneficial results on DSS-induced colitis mice when administered in a diet composed of AES at 25%. Indeed, the treatment with AES reduced the colon damage induced by hydrogen peroxide (H_2_O_2_) and hydroxyl radicals. AES improved the length of the colon and the structure of the crypts in the mucosal layer, and it showed high O_2_**·**^−^ radical-scavenging activity [23].

The overproduction of pro-inflammatory cytokines and chemokines is associated with the activation of phosphodiesterase 4 (PDE4), an enzyme involved in the activation and infiltration of immune cells into the inflamed tissue [89]. Bagalagel A. et al. (2022) demonstrated that the treatment of UC rats with a dose equivalent to 150 mg/kg of fucoidan administered by oral gavage reduced the expression of PDE4 and that it had additional beneficial effects, such as restoration of the normal shape of the mucosa and submucosa, as well as a normal length and weight of the colon [22]. Moreover, fucoidan treatment of acetic acid-induced UC mice attenuated both gene and protein expression of AhR, which is reduced or altered during gut inflammatory responses [90], leading to inactivation of the epithelial barrier and overexpression of pro-inflammatory mediators. Another beneficial effect of fucoidan treatment is the increased expression of the antioxidant transcription factor Nrf2, whose target gene is responsible for the release of heme oxygenase 1 (HMOX1) [28]. This increased expression resulted in lower levels of MDA, peroxynitrite (ONOO^−^) and GSH, and higher levels of glutathione peroxidase (GPx) [22].

A recent study focused on the treatment of a DSS-induced acute colitis mouse model by the administration of dried pellet providing a dose of 400 mg/kg (body weight) of fucoidan extracts from *Macrocystis pyrifera*, a brown alga belonging to the genus *Laminaria*. The results of the study showed that fucoidan is able to induce a decrease in inflammatory cell infiltration in the intestinal mucosa, suggesting a possible blockage of macrophage maturation and activation, as demonstrated by a significant reduction in cytokine levels, including TNF-α, IL-1β and IL-6 levels, as well as a decrease in IFN-γ, IL-1α, IL-3, IL-9, and IL-12. The results also showed an antioxidant activity after fucoidan treatment, considering the reduction of factors involved in oxidative stress, such as the levels of MDA, MPO, NO, SOD and CAT. In addition, *Macrocystis pyrifera* extract reduced the weight and length of the colon and the weight of spleen [24].

Another interesting compound with anti-inflammatory activity in IBD is eckol (Figure 2a), a phlorotannin characterized by a dibenzo-1,4-dioxin skeleton with a hydroxy group and an aromatic nucleus [91]. Eckol, isolated from the seaweed *Ecklonia cava*, has been shown to reduce the production of pro-inflammatory cytokines, such as TNF-α, IL-1β, and IL-6, while it can increase IL-10 levels in both serum and colon tissues of DSS-induced colitis in mice (oral administration of doses of 0.5–1.0 mg/kg of the body weight). Furthermore, eckol inhibits the NF-κB signaling pathway, suggesting it can exert an influence on the release of cytokines and chemokines. In addition, eckol showed general beneficial effects on gut health, reducing colorectal shortening by 14.3% and alleviating diarrhea and hematochezia [27].

DSS-induced colitis symptoms are also ameliorated by the administration of ethanoic extract (12 mg/kg of the body weight) of *Sargassum macrocarpum* (MES), rich in mero-terpenoids composed of sargahydroquinoic acid, sargachromeol, and sargaquinoic acid. Its main anti-inflammatory effects are the reduction of inflammatory cell infiltration, as evidenced by the reduction of MPO activity and, consequently, a significant effect in lowering the levels of pro-inflammatory cytokines, including TNF-α, IFN-γ, IL-1β, and IL-17. Moreover, MES can decrease the expression of MMP-2, -9, and -13, as well as NF-κB. Consistent with these findings, MES also normalized the iNOS levels and ameliorated the lesions of the colon in DSS-induced mice [29].

Yamada et al. (2014) illustrated the anti-inflammatory and anti-apoptotic effects of the seaweed *Dictyopteris undulata*. In particular, they reported the ability of zonarol, a marine hydroquinone extracted from brown alga (Figure 2c), to reduce inflammatory responses in the colonic epithelium of DSS-induced colitis mice when administered at doses of 10 and 20 mg/kg orally. Indeed, zonarol decreased the levels of TNF-α, IL-6, iNOS, and NO; reduced the hematochezia; and ameliorated the colon shortening [30].

An interesting compound derived from brown algae is dieckol (Figure 2e), a phenolic hexamer, whose structure is formed by two molecules of eckol connected by an oxygen molecule. The role of dieckol has been investigated in DSS-induced colitis mice treated with doses of 5, 10, and 15 mg/kg of body weight, demonstrating it can down-regulate the expression of pro-inflammatory factors, such as TNF-α, IL-1β, p65, and COX-2, as well as MDA and MPO levels. Furthermore, dieckol suppressed DSS-induced colitis by enhancing the expression of Nrf2 and HMOX1 [28]. This effect was particularly interesting because HMOX1 exerts a cytoprotective effect by reducing oxidative stress [92].

The anti-inflammatory activity of brown algae in potential IBD therapies was also confirmed by *Turbinaria ornata* extract, which is composed of several compounds, among which steroids and sulfoquinovosyl monoacylglycerols stand out. Treatment of DSS-induced chronic colitis mice with 15 mg/kg of *Turbinaria ornata* extract significantly reduced TNF-α and COX-2 expression, as well as MPO activity, in the colitis samples. Meanwhile, the extract administration increased the release of the anti-inflammatory cytokine IL-10. The histopathological evaluation also showed positive effects on alleviating the shortening of the colon and the colon lesions after treatment with *Turbinaria ornata* [31].

### 4.4. Anti-Inflammatory Activity of Rhodophyta (Red Algae) Extracts

Red seaweed also contains compounds with notable gut health benefits, particularly anti-inflammatory effects. This was demonstrated in the study by Reddy S.M. et al. (2023), who investigated polysaccharides extracted from species of the genus *Spyridia*. The study found that these compounds were associated with inhibition of the protein denaturation process [93]. Further findings on the antioxidant activity of red seaweeds, such as *Palmaria palmata* and *Porphyra dioica*, showed that their lipid extract can inhibit COX-2 expression by more than 80% [94]. In addition, *Porphyra tenera* extract showed a slight reduction in histologic damage in mice with DSS-induced colitis treated with 500–1000 mg/kg of algal extract and significantly decreased the mRNA levels of inflammatory markers in the colon, including TNF-α, IL-1β, IL-6, and COX-2. The study results presented by Kim et al. indicated that the *Porphyra tenera* extract positively impacted various aspects of colitis, such as mitigating body weight loss, preserving colon length, and improving the disease activity index (DAI) score in affected mice. In addition, the extract effectively alleviated DSS-induced inflammation by enhancing antioxidant activities in colitis mice and by modifying the gut microbiota composition in DSS mice. These positive results are related to the high number of polyphenols in the *Porphyra tenera* extract. Notably, the treatment enhanced beneficial intestinal metabolic pathways predicted based on gut microbial communities, including those related to glycine betaine metabolism [32], which is significant because many studies have reported that betaine administration reduces inflammatory markers such as TNF-α, IL-6, and iNOS [95].

In addition, a lipidomic study of *Grateloupia turuturu* identified the polar lipid profile of this red seaweed and demonstrated the presence of PUFAs, including eicosapentaenoic acid. The extracted polar lipids showed antioxidant and anti-inflammatory activities. In particular, *Grateloupia turuturu* extract, evaluated at five concentrations ranging from 12.5 to 250 μg mL^−1^, resulted in a 50% decrease in COX-2 levels at a lower dose than lipids from *Palmaria palmata* and *Porphyra dioica* [33].

Aquamin^®^ (Marigot Ltd., Cork, Ireland) was developed from the compounds isolated from the marine red algae belonging to *Lithothamnion* sp. Its composition is based on substances extracted from the skeletal remains of *Lithothamnion* algae, which are rich in calcium, magnesium and other elements. The efficacy of Aquamin^®^ has been tested on organoids treated with LPS and pro-inflammatory cytokines in order to compromise the gastrointestinal barrier function. Based on the most recent study, the treatment with Aquamin^®^ improved barrier function by downregulating proteins involved in pro-inflammatory pathways, while upregulating those with anti-inflammatory, antioxidant and antimicrobial activities [96].

## 5. Conclusions

Under physiological conditions, the inflammatory response provides a vital biological defense against tissue damage and infection, with the goal of restoring the body’s homeostasis. This process is typically self-limiting and rapidly terminates when the underlying cause of inflammation, such as an infection, is effectively treated. IBD has become a major area of research in recent years due to its serious consequences [97,98,99,100]. IBD is a chronic disease that requires continuous medication. While conventional treatments such as 5-ASA, corticosteroids, immunosuppressants and antibiotics are effective in treating IBD, they are associated with significant side effects and the risk of drug resistance with prolonged use.

The persistent nature of this disease means that while initial treatments may provide some benefit, patients often experience serious side effects or diminished therapeutic responses over time due to continuous drug administration. The situation is further complicated by the growing problem of bacterial resistance to traditional synthetic antibiotics, prompting researchers to seek alternative approaches. This has led to a great interest in exploring natural products for the treatment of IBD.

**Figure 3:**
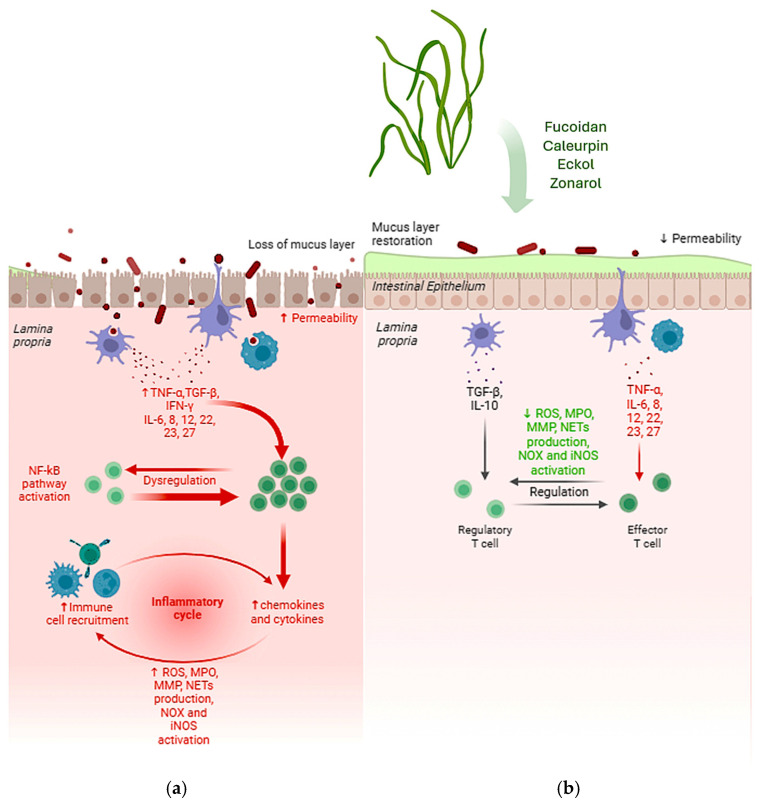
Main effects of bioactive compounds extracted from algae with anti-inflammatory effects. The typical gut affected by IBD produces less mucus, the intestinal epithelium shows increased permeability and the dysregulation of immune cell recruitment leads to increased levels of chemokines and cytokines, which cause a chronic inflammatory response (**a**). On the other side, after treatment with algal extracts, the mucus layer is restored, reducing permeability and restoring the normal regulation of the inflammatory response, showing decreased production of ROS, MPO, MMP and NETs, as well as the decreased NOX and iNOS activation (**b**). ↑ = increased; ↓ = decreased.

Recently, compounds derived from marine sources, particularly algae, have emerged as a promising area of research. These algae are known to produce a number of bioactive secondary metabolites that exhibit significant anti-inflammatory properties (Figure 3), making them potential candidates for therapeutic use. In light of this growing field of study, this review illustrated the anti-inflammatory potential of algal-derived biomolecules, specifically in the context of IBD and gut health. The preliminary results of the review indicate that a large number of algae possess a remarkable therapeutic potential for the treatment of IBD, mainly due to the anti-inflammatory and antioxidant activities of their bioactive compounds. However, further research is needed to assess how these extracts compare to standard treatments. A key challenge remains the lack of in-depth understanding of how the specific structural features of algal polysaccharides influence their biological activity. Future research should focus on elucidating the cellular and molecular mechanisms underlying the effects of algal-derived compounds. Clinical trials are essential to determine the safety and efficacy of algal extracts in the treatment of IBD, and additional in vivo studies, particularly in humans, are needed to better understand and confirm the potential of algal extracts in the prevention of IBD.

## Figures and Tables

**Table 1:** Algae anti-inflammatory effects demonstrated in vivo and relevant compounds extracted. ↑ = increased; ↓ = decreased.

Algae	Phyla	In Vivo Models	Main Effects	Relevant Compounds	Concentration	Reference
*Chlorella vulgaris*	*Chlorophyta*	DSS-induced colitis	↑ Regulatory T cells	Not investigated	2 g/kg	[19]
*Caulerpa racemosa*	*Chlorophyta*	DSS-induced colitis	↓ TNF-α, IFN-γ, IL-6, IL-17, NFκB p65 ↑ IL-10	Caulerpin	40–4 mg/kg	[26]
*Ulva pertusa*	*Chlorophyta*	DNBS-induced colitis	↑ COX-2, iNOS, IL-4 ↓ NO, SOD, CAT, GSH, MDA, NF-κB signaling pathway	Not investigated	10–50–100 mg/kg	[20]
*Ulva pertusa*	*Chlorophyta*	DNBS-induced colitis	↓ IL-6, IL-17, IL-23 ↑ IL-10	Not investigated	50–100 mg/kg	[21]
*Laminaria* genus	*Phaeophyta*	Soybean-induced intestinal inflammation	↓ Expression of inflammatory response-related genes	Alginate oligosaccharides, β-(1, 3)-glucan	5% of the body weight	[25]
*Undaria pinnatifida*	*Phaeophyta*	DNBS-induced colitis	↓ H_2_O_2_, hydroxyl radicals ↑ •O^−2^ radical-scavenging activity	Fucoidan	25% of the diet	[23]
Not mentioned	*Phaeophyta*	Acetic acid-induced UC	↓ PDE4, MDA, ONOO^−^, GSH ↑ AhR, Nrf2, HMOX1, GPx	Fucoidan	150 mg/kg	[22]
*Macrocystis pyrifera*	*Phaeophyta*	DSS-induced colitis	↓ TNF-α, IL-1β, IL-6, IFN-γ, IL-1α, IL-3, IL-9, IL-12, MDA, MPO, NO, SOD, CAT	Fucoidan	400 mg/kg	[24]
*Ecklonia cava*	*Phaeophyta*	DSS-induced colitis	↓ TNF-α, IL-1β, IL-6, NF-κB signaling pathway ↑ IL-10	Eckol	0.5–1.0 mg/kg	[27]
*Sargassum macrocarpum*	*Phaeophyta*	DSS-induced colitis	↓ MPO, TNF-α, IFN-γ, IL-1β, IL-17, MMP-2, MMP-9, MMP-13, NF-κB signaling pathway, iNOS	Sargahydroquinoic acid, sargachromeol, sargaquinoic acid	12 mg/kg of the body weight	[29]
*Dictyopteris undulata*	*Phaeophyta*	DSS-induced colitis	↓ TNF-α, IL-6, iNOS, NO	Zonarol	10–20 mg/kg	[30]
Not mentioned	*Phaeophyta*	DSS-induced colitis	↓ TNF-α, IL-1β, p65, COX-2, MDA, MPO ↑ Nrf2, HMOX1	Dieckol	5–10–15 mg/kg of body weight	[28]
*Turbinaria ornata*	*Phaeophyta*	DSS-induced colitis	↓ TNF-α, COX-2, MPO ↑ IL-10	Steroids and sulfoquinovosyl monoacylglycerols	15 mg/kg of body weight	[31]
*Porphyra tenera*	*Rhodophyta*	DSS-induced colitis	↓ TNF-α, IL-1β, IL-6, COX-2 ↑ Glycine betaine metabolism-related gut bacteria	Polyphenols	500–1000 mg/kg of body weight	[32]
*Grateloupia turuturu*	*Rhodophyta*	Not investigated	↓ COX-2	PUFAs	Five concentrations ranging between 12.5 and 250 μg mL^−1^	[33]
